# Active remote-site musculoskeletal infection as a risk factor for periprosthetic infection in a new joint implant: A case series

**DOI:** 10.1016/j.amsu.2019.07.013

**Published:** 2019-07-11

**Authors:** Bassem I. Haddad, Jihad Alajlouni, Mohammad Hamdan, Ala' Hawa, Elsiddig E. Mahmoud

**Affiliations:** a Faculty of Medicine, Special Surgery Department, Orthopaedics and Trauma Department, University of Jordan, Queen Rania Street, Amman, 11942, Jordan; b Faculty of Medicine and Health Sciences, Omdurman Islamic University, P. O. Box 382, Omdurman, Sudan

**Keywords:** Arthroplasty, Remote-site infection, Periprosthetic joint infection, Musculoskeletal, Contraindication, CRP, C-reactive protein, DHS, dynamic hip screw, ESR, erythrocyte sedimentation rate, PJI, periprosthetic joint infections

## Abstract

**Introduction:**

Arthroplasty has always been associated with complications, such as the possibility of periprosthetic infection. The presence of an active infection at the site of the planned surgery is considered a contraindication for the new implant. However, it is unclear whether there is an association between the presence of remote musculoskeletal infection and the development of infection in the prosthetic joint itself. We report six cases involving patients with active ongoing musculoskeletal infections at a remote site who underwent arthroplasty.

**Presentation of cases:**

Four male and two female patients were included in this review. Three patients underwent total hip arthroplasty, one underwent hip hemiarthroplasty, and two underwent total knee arthroplasty. All surgeries were performed in the presence of different stages of infection at a remote site; two had active infections with pus-discharging sinus, one was being treated with long-term oral antibiotic suppression, and three patients were diagnosed with remote prosthetic joint infections on the basis of joint aspiration or intraoperative cultures. Clinical assessments of pain, wound erythema or drainage, and soft tissue swelling were performed at follow-up. Radiography and analysis of inflammatory marker levels were performed preoperatively and 6 weeks postoperatively.

**Discussion:**

All six patients were followed-up for at least 18 months (mean, 4.6 years; range, 18 months to 9 years). No evidence of superficial surgical-site infection or deep prosthetic joint infection was observed.

**Conclusion:**

The presence of an active infection at a remote site might not be a contributing factor to periprosthetic joint infection.

## Highlights

• Six patients underwent arthroplasty in presence of active remote-site infections.
• No evidence of superficial surgical-site infection was seen (18-month follow-up).
• No evidence of deep prosthetic joint infection was observed (18-month follow-up).
• Active remote-site infection is not associated with periprosthetic joint infection.

## 1 Introduction

Periprosthetic joint infections (PJI) are one of the common complications following joint arthroplasty. They have devastating effects on the outcome of joint replacement surgery, with a heavy burden on the patient, surgeon, and hospital.

As the number of joint arthroplasties being performed worldwide is constantly increasing [[1], [2], [3], [4]], surgeons are often faced with the need to perform joint arthroplasty on patients with an ongoing musculoskeletal infection at another site.

Ongoing infections, especially those of the urinary tract or dental infections, are reported to be a risk factor for PJI following joint arthroplasty [[5], [6], [7]]. However, the link between the presence of remote musculoskeletal infections and the development of PJI remains unclear.

We report six cases of patients with active ongoing musculoskeletal infections at a remote site who underwent arthroplasty. All cases have been reported in line with the PROCESS criteria [8].

## 2 Presentation of cases

We retrospectively reviewed the data of six patients who had a chronic musculoskeletal infection and underwent joint arthroplasty surgeries during the active phase of infection. All these patients underwent the new joint implant surgeries for a fracture or significant disability. Five of the new joints were implanted at our institute, the last joint was implanted outside our institute, but the patient was followed at our department.

The study was approved by the appropriate Institutional Review Board. Informed written consent was obtained from the patients after explaining the primary aims of the study and ensuring the confidentiality of obtained information. All patients who underwent surgery our hospital provided written informed consent regarding the risk of periprosthetic infection following the new implant. Culture-specific intravenous antibiotics were continued for 6 weeks postoperatively under the supervision of an infectious disease specialist.

The follow-up period ranged from 18 months to 9 years (mean 4.6 years), during which patients were clinically assessed for pain and wound erythema or drainage. Radiographs were assessed for signs of loosening. Inflammatory marker levels (erythrocyte sedimentation rate [ESR] and C-reactive protein [CRP]) were analyzed preoperatively and 6 weeks postoperatively. None of the patients showed clinical or radiological evidence of infection in the new implant.

### 2.1 Case (1)

**Fig. 1:**
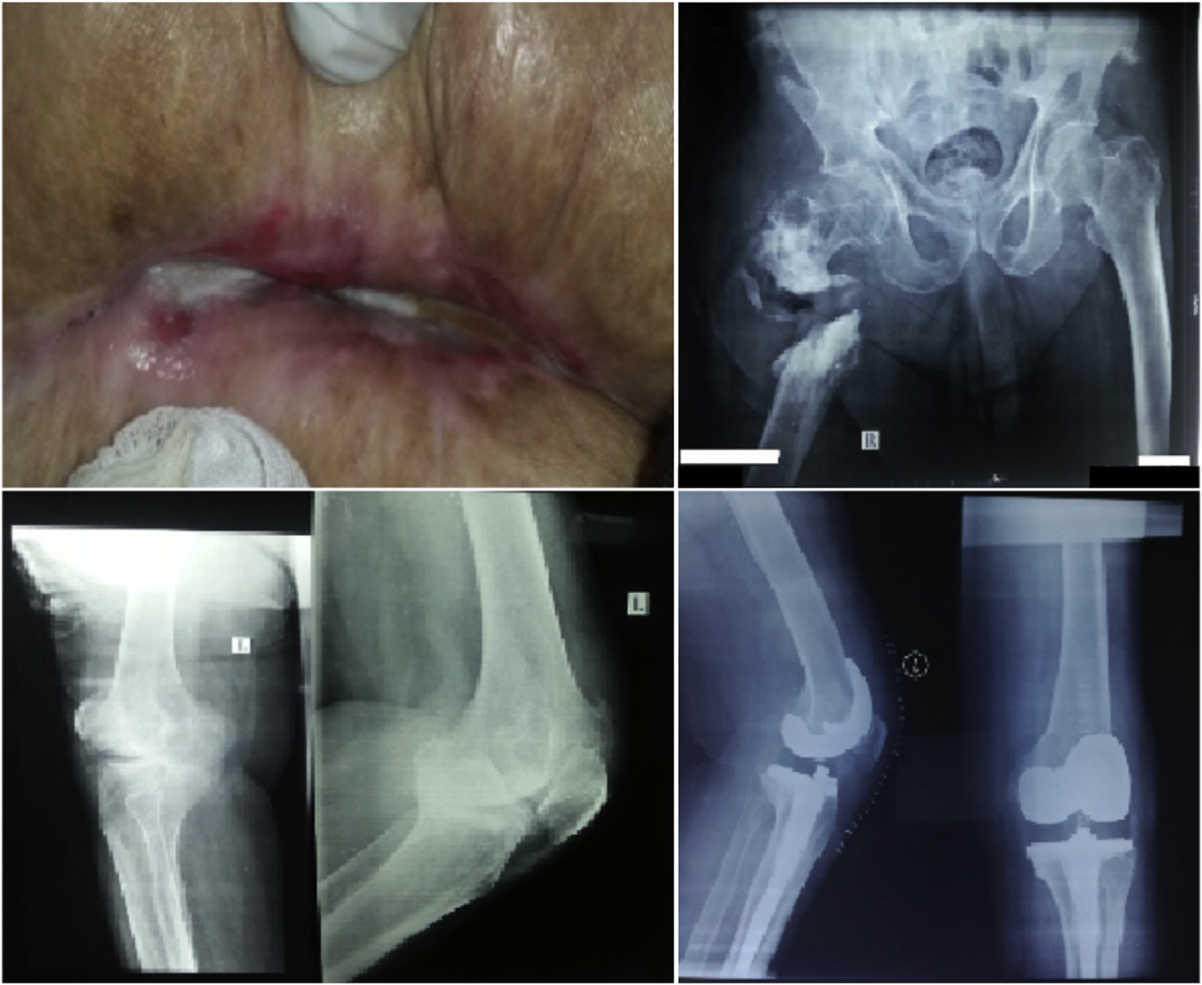
Clinical photo and X-ray image of the infected right femur, and preoperative and postoperative X-ray images of the left knee.

A 65-year-old diabetic male patient presented with infected nonunion of a right femur fracture with a draining sinus. The patient had disabling advanced left knee osteoarthrosis. Conservative treatment was unsuccessful, so left total knee arthroplasty was performed in September 2014 (Fig. 1).

### 2.2 Case (2)

A 65-year-old diabetic male patient, who underwent left total knee arthroplasty in 2010 outside of our hospital, suffered early periprosthetic infection and was treated with long-term antibiotic suppression. In 2011, the patient sustained a traumatic ipsilateral femur neck and periprosthetic knee fracture. He underwent uncemented hip bipolar hemiarthroplasty, and open reduction and internal fixation of the distal femur outside our hospital.

**Fig. 2:**
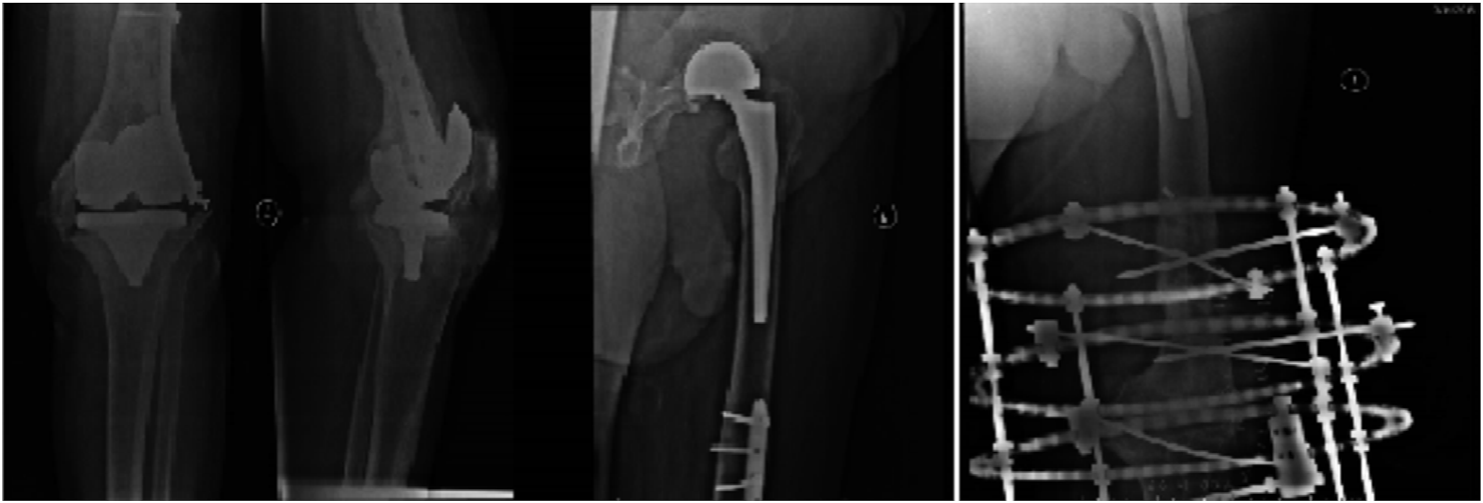
X-ray of the fixation of the periprosthetic fracture of the infected loose left knee prosthesis, also showing the ipsilateral uncemented hip bipolar prosthesis. Knee arthrodesis by Ilizarov external fixator was performed later.

In March 2016, the patient presented with a flare-up of the left knee infection, X-ray images revealed loosening of the prosthesis. Bone scintigraphy indicated that infection was localized to the knee prosthesis and did not involve the hip prosthesis. Knee arthrodesis was performed (Fig. 2).

### 2.3 Case (3)

**Fig. 3:**
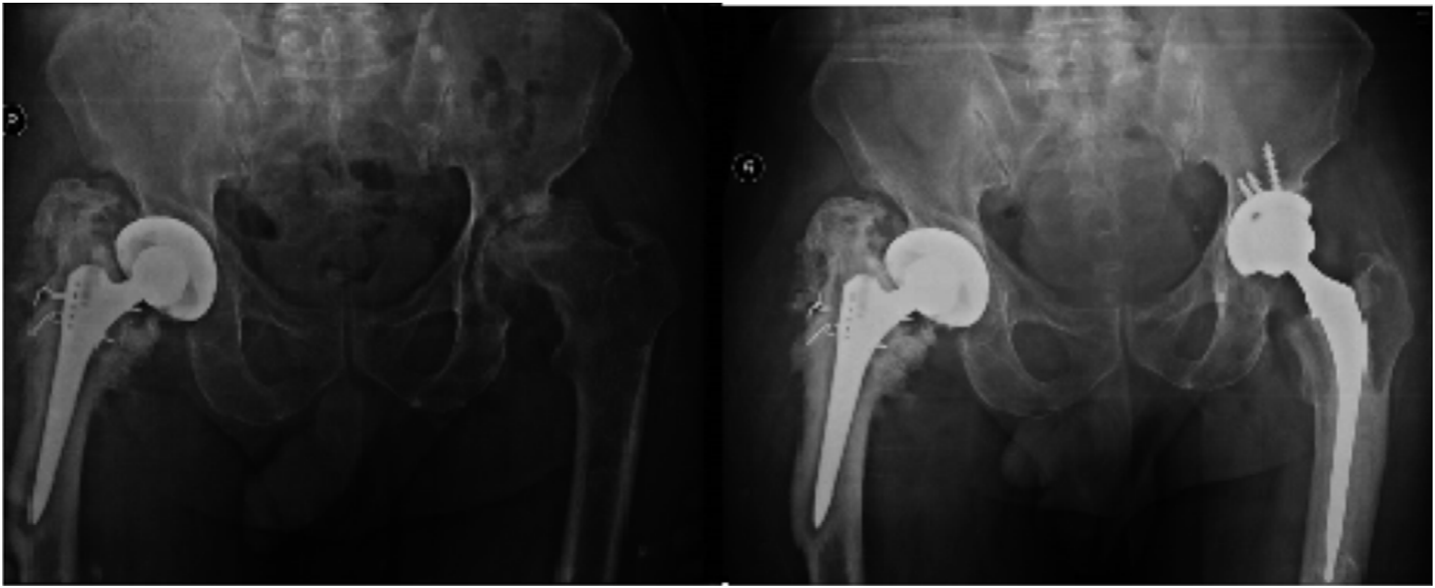
Preoperative and postoperative X-ray images for left total hip arthroplasty performed for severe hip osteoarthrosis. The loose, infected right hip prosthesis is evident.

A 73-year-old healthy man presented in 2015 complaining of disabling advanced left hip osteoarthrosis and an infected right total hip arthroplasty, which was performed and managed with antibiotic suppression outside our hospital. Due to the significantly limited patient's functional status, left total hip arthroplasty with a cemented stem and uncemented cup was performed in January 2016 (Fig. 3). A two-stage revision of the infected right hip was performed later.

### 2.4 Case (4)

**Fig. 4:**
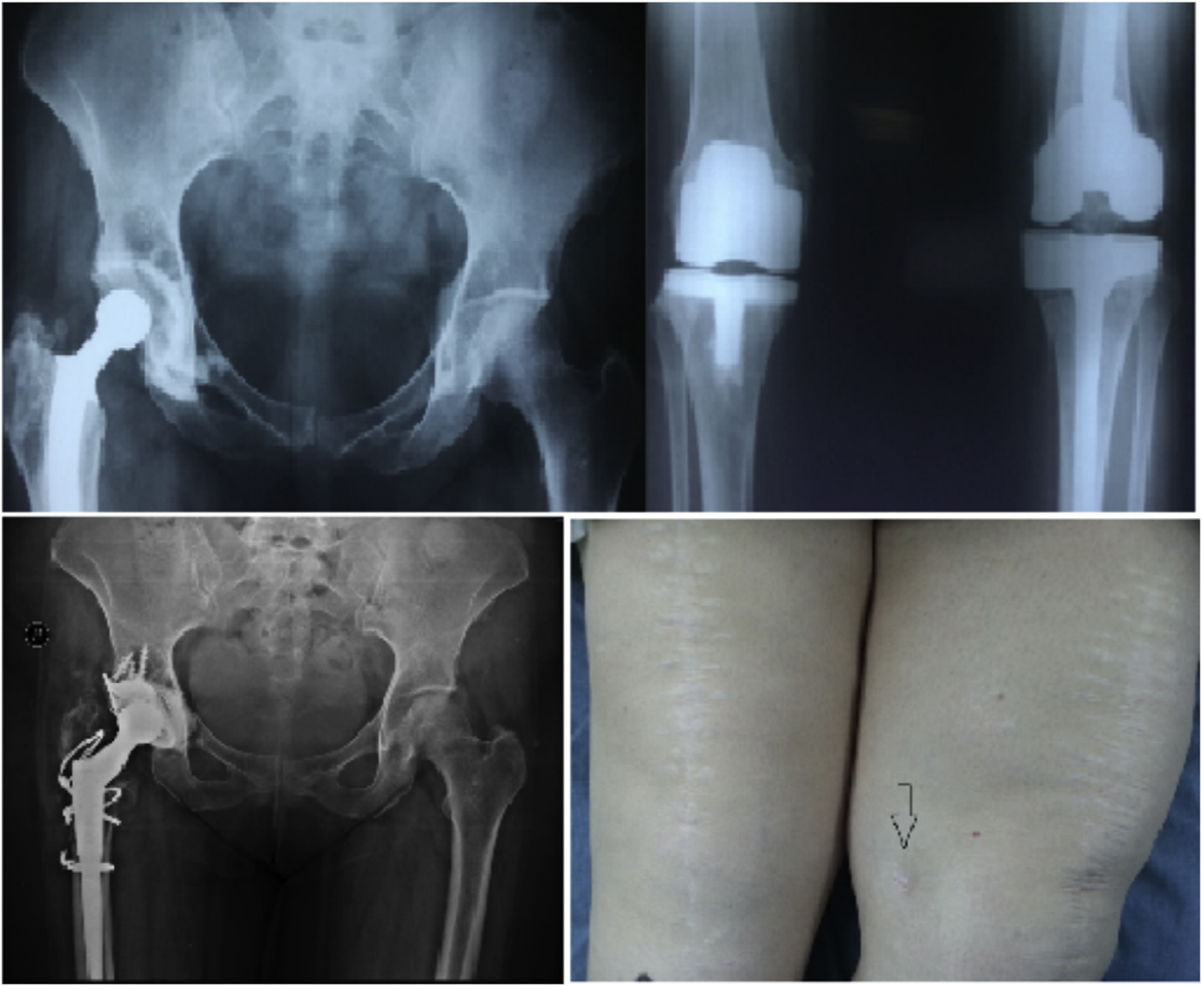
Preoperative X-ray image showing bilateral knee prostheses and right hip prosthesis and postoperative X-ray image of the revision hip prosthesis performed in the presence of a discharging sinus in the left knee (arrow).

A 55-year-old woman with a history of hypertension and rheumatoid arthritis underwent bilateral total knee replacement and right total hip replacement in 1994 outside our hospital. She presented with left knee discharging sinus which was treated with long-term culture-directed antibiotic therapy. During follow-up, the patient developed disabling right hip pain resulting from aseptic loosening of the total hip prosthesis (infection was ruled out by hip aspirate). Revision total hip arthroplasty was performed in May 2015 (Fig. 4).

### 2.5 Case (5)

A 76-year-old healthy woman with severe bilateral hip osteoarthrosis presented with a 6-month history of two-stage revision for infected right total hip prosthesis. No signs of active infection were apparent. In October 2009, she underwent left total hip arthroplasty at our hospital. During follow-up, the patient complained of pain and pus discharge from the right hip. The infection was treated by excision arthroplasty of the right hip. The left hip remained asymptomatic.

### 2.6 Case (6)

**Fig. 5:**
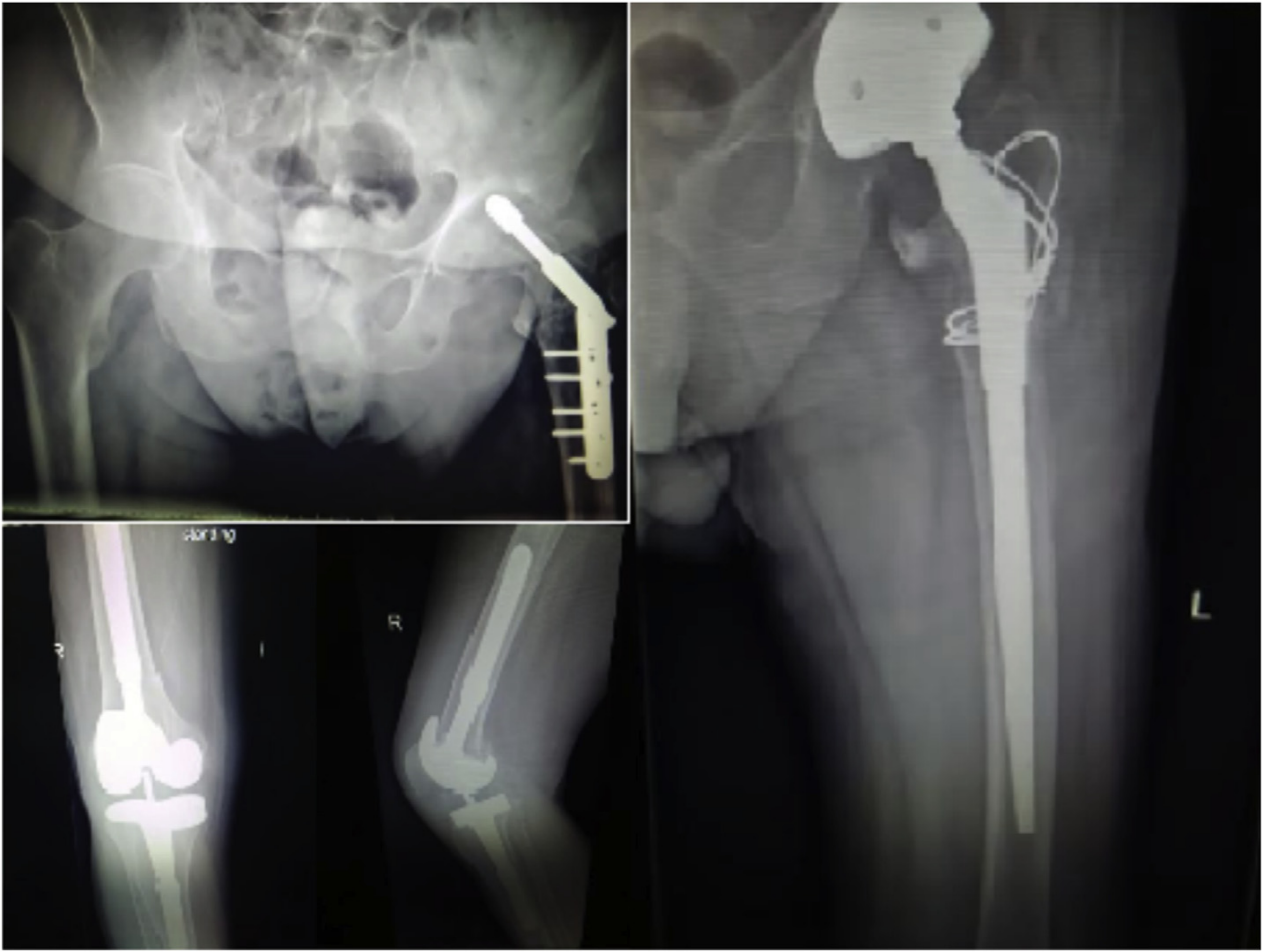
X-ray images showing the dynamic hip screw cut-out from the left hip and the totally asymptomatic right knee prosthesis and postoperative left hip image.

A 75-year-old healthy man underwent left dynamic hip screw (DHS) fixation for a hip fracture in October 2016 and right total knee replacement for primary osteoarthrosis in May 2017 outside of our hospital. He presented to our clinic in November 2017 complaining of left hip pain due to screw cut-out and erythema over the surgical site. The DHS was removed, cultures were taken, and total hip replacement carried out at the same setting. Intra-operative cultures were positive and postoperative IV antibiotics were continued for 6 weeks. The right knee remained asymptomatic (Fig. 5).

## 3 Discussion

Hip and knee arthroplasties are common in orthopedic practice, with around 700,000 total hip and knee arthroplasties performed yearly in the US alone [4]. According to Maradit et al. [9], around 7.2 million Americans were living with hip or knee prostheses in 2010. The revision burden of these surgeries (defined as the number of revision arthroplasties performed during a year compared with the total number of arthroplasties performed in the same year) averaged around 10% for hip arthroplasties and 8% for knee arthroplasties from different registries worldwide, according to the American Joint Replacement Registry 2016 annual report [10]. This report also states that PJI accounted for 8.4% of overall hip revision indications, and 18.4% of early hip revision procedures (performed <3 months after the primary procedure). PJI accounted for 9.3% of overall knee revision indications and 44.8% of early knee revision procedures (performed <3 months after the primary procedure) [10].

Classically, the presence of active ongoing infection in the body is considered a contraindication for arthroplasty. Numerous studies have discussed the risk of PJI due to remote active infections. However, these studies assessed the risk of developing PJI in patients who developed a remote infection with the prosthesis in situ, rather than the risk of PJI in patients who already have an ongoing musculoskeletal infection and are undergoing hip or knee arthroplasty.

In a prospective cohort study, occurrences of PJI associated with remote infections were concluded to be rare [11]. Lim et al. reported two cases of implant infection with *Staphylococcus aureus* that were associated with dermatitis, and they concluded that dermatitis at sites remote from the operative site may be a contributing factor to implant infection [12]. Sendi et al. carried out a retrospective cohort study of patients with in situ prostheses who developed *Staphylococcus aureus* bacteremia and discovered that 30% of these patients also developed PJI [13]. However, many studies have found no association between PJI and the presence of asymptomatic urinary tract infection at the time of surgery, and the benefit and cost effectiveness of urine cultures and preoperative antibiotic treatment in patients with asymptomatic bacteriuria have been questioned [[14], [15], [16], [17]].

To our knowledge, only two reports provide insight into the risk of PJI in prostheses that were implanted in the presence of active remote musculoskeletal infections [18,19]. Cherney and Amstutz in 1983 [18] reported a 30% incidence of periprosthetic infection in 33 hips, which were operated on in the presence of active sepsis. In Jupiter's analysis of 57 hips [19], 18 had an active infection at the time of arthroplasty, of which 14 arthroplasties had no evidence of infection at a mean follow-up time of 42 months. However, these patients had infections at the site of surgery rather than remotely. In another study [20], 55 patients with multiple prosthetic joints were followed after developing PJI. It was found that 20% developed an infection in another prosthetic joint after an average of 2 years.

In the present study, we report six cases in which arthroplasties were performed in the presence of an active infection at a remote musculoskeletal site (PJI or chronic osteomyelitis). All patients were treated with culture-specific intravenous antibiotics for 6 weeks postoperatively (Table 1). They were followed for a mean duration of 4.6 years (range: 18 months to 9 years). During that period, patients were assessed clinically, radiographically, and by laboratory studies (namely, ESR and CRP levels). None of the patients showed any evidence of superficial surgical site infection or deep PJI (Table 2).

**Table 1:** Preoperative and postoperative characteristics of patients.

Patient	Comorbidities	ASA score	Operation site	Infected side	Microorganism	Antibiotics	Pre-op ESR/CRP mm⋅Hr^−1^/mg⋅L^−1^	Post-op ESR/CRP (6 weeks) mm⋅Hr^−1^/mg⋅L^−1^
1	DM	2	Left knee	Right femur	Polymicrobial	Imipenem, vancomycin	110/31	53/18
2	DM	2	Left hip	Left knee	Coagulase -ve *S. aureus* (CONS)	Imipenem, vancomycin	95/39	80/20
3	--	1	Left hip	Right hip	(CONS)	Imipenem, vancomycin	130/44	75/18
4	HTN, RA	2	Right hip	Left knee	(CONS)	Ciprofloxacin, clindamycin	48/40	30/4
5	--	1	Left hip	Right hip	Polymicrobial	Vancomycin, cefuroxime	115/117	77/16
6	--	1	Right knee	Left hip	*Pseudomonas*	Imipenem, vancomycin	26/4	30/2

Patients are numbered as described in the case study section.

Abbreviations: ASA, American Society of Anesthesiologists; CRP, C-reactive protein; DM, diabetes mellitus; ESR, erythrocyte sedimentation rate; HTN, hypertension; RA, rheumatoid arthritis.

**Table 2:** Duration and results of patient follow-up.

Patient	Follow-up duration and results
1	Period = 4 years, no clinical or radiological findings of infection in the left knee
2	Period = 7 years, no clinical or radiological findings of infection in the left hip
3	Period = 2 years and 9 months, no clinical or radiological findings of infection in the left hip
4	Period = 3.5 years, no clinical or radiological findings of infection in the right hip
5	Period = 9 years, no clinical or radiological findings of infection in the left hip
6	Period = 18 months, no clinical or radiological findings of infection in the right knee

Patients are numbered as described in the case study section.

The presence of active infection is a deferring factor for most orthopedic surgeons. In this small study, patients’ conditions necessitated these procedures. The absence of PJI development in the new implants suggests that the presence of active musculoskeletal infection at a remote site might not be associated with the increased risk of development of infection in the new implant. The long follow-up period strengthens the results of this study, which provides insight into a subject that is not been well represented in the literature. A limitation of this study is the very small number of patients.

## 4 Conclusion

The presence of remote musculoskeletal infection might not be a contributing factor to the development of periprosthetic infection in a new joint implant. Further studies are required to definitively establish this relationship.

## Provenance and peer review

Not commissioned, internally reviewed.

## Funding support

None.

## Ethical approval

The study was approved by Institutional Review Board (IRB) at Jordan University Hospital, Amman, Jordan, session number 6/2016-2017, on 28/2/2017, reference number 703/2017/67. Informed written consent was obtained from the patients after explaining the primary aims of the study and ensuring the confidentiality of obtained information.

## Sources of funding

None.

## Author contribution

Bassem I. Haddad: Writing and revising the manuscript.

Jihad Alajlouni: Senior surgeon who performed the surgeries, conception of the idea, patient follow-up.

Mohammad Hamdan: Writing and editing the manuscript.

Ala’ Hawa: Medical records review, literature review.

Elsiddig Mahmoud: Medical records review, literature review.

## Research registry number

www.researchregistry.com.http://www.researchregistry.com/

UIN: researchregistry4694.

## Guarantor

Bassem I. Haddad.
